# Nestling activity levels during begging behaviour predicts activity level and body mass in adulthood

**DOI:** 10.7717/peerj.566

**Published:** 2014-09-16

**Authors:** Luke S.C. McCowan, Simon C. Griffith

**Affiliations:** Department of Biological Sciences, Macquarie University, Sydney, NSW, Australia

**Keywords:** Begging, Activity, Personality, Ontogeny, Behavioural consistency, Zebra finch, Developmental biology, Begging movements

## Abstract

Across a range of species including humans, personality traits, or differences in behaviour between individuals that are consistent over time, have been demonstrated. However, few studies have measured whether these consistent differences are evident in very young animals, and whether they persist over an individual’s entire lifespan. Here we investigated the begging behaviour of very young cross-fostered zebra finch nestlings and the relationship between that and adult activity levels. We found a link between the nestling activity behaviour head movements during begging, measured at just five and seven days after hatching, and adult activity levels, measured when individuals were between three and three and a half years old. Moreover, body mass was found to be negatively correlated with both nestling and adult activity levels, suggesting that individuals which carry less body fat as adults are less active both as adults and during begging as nestlings. Our work suggests that the personality traits identified here in both very young nestlings and adults may be linked to physiological factors such as metabolism or environmental sources of variation. Moreover, our work suggests it may be possible to predict an individual’s future adult personality at a very young age, opening up new avenues for future work to explore the relationship between personality and a number of aspects of individual life history and survival.

## Introduction

When the behaviour displayed by individuals in a population or species is characterised by both inter-individual variation and intra-individual consistency over time and/or context, this infers the existence of personality differences between individuals (Carere & Maestripieri, 2013; Réale et al., 2007). One example of a commonly measured personality trait, which has been established and measured in a range of species, is activity (Beauchamp, 2000). ‘Activity’ can be defined as the general activity level of an individual, usually measured in an environment that is not novel or risky to reduce interference with other personality traits such as exploration or boldness (Réale et al., 2007). Activity has often been shown to be repeatable when measured across lengths of time that make up a considerable portion of an individual’s lifespan (Herde & Eccard, 2013; Martin & Réale, 2008). Moreover, several studies have shown that activity is repeatable over different life stages from ontogeny to adulthood (Kanda, Louon & Straley, 2012).

In young nestling birds, individuals may vary in their levels of activity, and this may influence their interactions with both their parents and siblings in the nest, thereby influencing the amount of food they receive and their growth patterns, although high activity levels may have an energy cost (Roulin, 2001; Gilby, Mainwaring & Griffith, 2011). Begging in young birds is made up of a number of separate components which are often performed at the same time during a begging bout, such as auditory calling, raising of the head, body posture, bill gaping, pecking and approaching the parent, and movements of the head and tongue (Eising & Groothuis, 2003; Gilby, Sorato & Griffith, 2012; Kacelnik et al., 1995; Ruuskanen & Laaksonen, 2013). In the zebra finch, *Taeniopygia guttata*, the mean intensity with which nestlings produce several of these different begging behaviours has been shown to be positively correlated, e.g., the rate of head movements is positively correlated with the rate of tongue movements (Gilby, Sorato & Griffith, 2012). Differences in begging behaviour between individuals may result from nestlings adapting to the personality of their parents, but they may also provide indications of underlying individual differences in their own personality, which may have been shaped by genetic differences or maternal effects such as the allocation of hormones during ontogeny (Roulin, Dreiss & Kölliker, 2010; von Engelhardt et al., 2006).

In the zebra finch, nestlings shake their heads from side to side and produce lateral movements of their tongues in their gaping mouths during begging, with the mean rate of lateral tongue movements increasing with age (Muller & Smith, 1978; Zann, 1996). Head and tongue movements attract the attention of the parents, soliciting provisioning and enable effective sibling competition for food (Kilner & Johnstone, 1997). The overall level of begging displayed by nestlings is linked to offspring need and usually related to the amount of food an individual receives from its parents (Gilby, Mainwaring & Griffith, 2011; Kilner & Johnstone, 1997). However, in zebra finches there is no significant correlation between the mean rate of tongue movements made by a nestling and the frequency that this nestling is fed by the parents (Muller & Smith, 1978), negating the idea that nestlings increase their tongue movement speed in order to receive more food. Hence, the speed of these movements during begging bouts (shown by their mean rate during a bout) may be more reflective of an individual’s general activity levels. Conversely, other begging variables, such as the duration of begging, gape width and the latency to start begging, while correlated with tongue and head movements, may be more closely linked to nutritional need, and predictive of food received than a nestling’s general activity level. Moreover, typical begging traits such as gape width and latency to start begging do not measure repetitive movement behaviours, such as those typically measured in traits which are the target of personality studies of activity in adults (David, Auclair & Cézilly, 2011).

Previous evidence that begging intensity may be reflective of an individual’s personality, or their coping style on the proactive-reactive axis, has been shown by studies featuring individuals of differing selection lines (Carere, 2003; Huff et al., 2007). Moreover, several non-begging nestling behaviours, namely handling aggression, docility and breathing rate (Brommer & Kluen, 2012; Fucikova et al., 2009), along with movements in a novel environment in young birds, may constitute personality traits (Ruuskanen & Laaksonen, 2010; Vennerholm, 2012). Several other studies have found that certain behavioural traits are repeatable across life stages when measured in juveniles and again as adults, such as exploratory behaviour in great tits (Carere et al., 2005b) and zebra finches (first measured at two months of age) (David, Auclair & Cézilly, 2012), and activity, neophobia, docility, social behaviour and exploratory behaviour in mammals (Cavigelli & McClintock, 2003; Herde & Eccard, 2013; Kanda, Louon & Straley, 2012; Petelle et al., 2013; Seyfarth, Silk & Cheney, 2012) and amphibians (Wilson & Krause, 2012). However, there is not always a correlation between personality measured in juvenile and adult stages (Herde & Eccard, 2013; Kanda, Louon & Straley, 2012; Wilson & Krause, 2012).

In this study, we investigated whether very early nestling mechanical behaviours produced during begging are related to adult personality in the zebra finch, a well-used model system for the study of parental care and personality. This study is the first to compare behavioural traits exhibited during food begging with personality much later in life. Specifically, we focused on those components of begging most likely to be indicative of general activity levels, namely tongue movements and head movements, and contrasted these with general activity levels measured in adults in a relatively low-stress environment, with a social partner in a familiar home cage. We predicted that nestling activity levels during begging would be positively correlated with their subsequent activity levels as adults.

## Methods

### General methods

Data were collected from January 2009 to July 2012 from domestic ‘wild-type’ zebra finches originally obtained from aviculturists in the Sydney region in 2005 and bred at Macquarie University for at least three generations (Tschirren et al., 2009). All subjects spent the majority of their lives under the same conditions in single or mixed-sex outdoor aviaries (8 × 10 × 2 m), with brief periods spent indoors in home wire cages (77 × 48 × 40 cm), holding one to four individuals while experiments were conducted (usually individuals were held in pairs or small social groups for social enrichment). In both environments, individuals were provided with ad libitum commercial finch seed and drinking water, along with grit, cuttlefish bone and a daily provision of chopped spinach and peas. Individuals were kept on a 11:13 h light:dark regime when housed indoors at a constant temperature of 24 °C and relative humidity maintained at around 40–50%. Individuals were subjected to the ambient conditions in Sydney, Australia when housed in outdoor aviaries.

### Nestling begging behaviour

All individuals were reared by domestic ‘wild-type’ foster parents between January and December 2009 in experimentally manipulated asynchronous broods (Gilby, Sorato & Griffith, 2012), produced by parents in an aviary (8 × 10 × 2 m) containing 20 males and 20 females. On the morning an egg was laid, it was replaced with an imitation egg, marked and placed in an artificial incubator until hatching. On the day of hatching, nestlings were individually-marked (by clipping a claw) and randomly cross-fostered to either a ‘control’ or ‘reverse’ experimental brood in the aviary (but never returned to the nest of their genetic parents) (Gilby, Sorato & Griffith, 2012). Each brood consisted of either five or six nestlings, placed into the nest in two groups of two to three nestlings 48 h apart. In ‘control’ broods, the first 2–3 nestlings placed into a nest were from position 1–3 of the laying sequence, and the latter 2–3 nestlings from position 4–6. In ‘reverse’ broods this pattern was reversed. In total there were 28 successful experimental broods containing 98 nestlings, 15 ‘control’ and 13 ‘reverse’, which came from 43 different genetic nests. When each nestling was five days old, and again at seven days old, it was removed from its nest and individually placed in an artificial nest on a heat pad to assay begging behaviour. Only a single nestling was taken from a nest at a time in random order, hence the time that each nestling was removed varied (range 11 a.m.–4 p.m.). Begging was measured in two rounds of three trials each day, with the first round beginning 15 min after the nestling was placed on top of a heat pad in the laboratory. To control for the variation in the level of hunger across different chicks (subject to recent parental allocation) we also measured begging a second time after a standardised feed and rest. Each chick was fed with liquefied egg and biscuit rearing and conditioning food mix until satiation and then left for one hour, before begging behaviour was again assayed. On each day the initial begging levels were strongly correlated with the latter ones (Pearson correlation: day 5: *r* = 0.77, *N* = 98, *P* < 0.0001; day 7: *r* = 0.76, *N* = 98, *P* < 0.0001; Gilby, Sorato & Griffith, 2012). Although this suggests that variation in begging behavior is not primarily determined by hunger alone, we nevertheless focus here on those measurements taken after the feeding procedure that standardized hunger levels across all chicks—and provided the best estimate of intrinsic variation in activity levels. During each begging measure, begging was initiated using a standardised procedure with a red pen to tap the nesting material around the nestling, the nestling’s beak and their left and right wing. This routine was conducted three times, with three minutes between each repeat. From video recordings, six different begging variables were quantified: Latency, Duration, Raised head, Head movements, Open mouth and Tongue movements. In total, all of the 98 nestlings (47 males and 51 females) completed the begging assays, producing a total of 568 observations. Further details of the procedures performed on the birds during this relatively early stage of development are described in Gilby, Sorato & Griffith (2012). Unfortunately, as three and a half years is roughly the average lifespan of the zebra finch in captivity (Heidinger et al., 2012), a large number of individuals had succumbed to old age by the start of the adult component of the work in 2012. Due to this natural attrition and the complex design of the cross-fostering experiment we did not have a sufficient sample size to explore the relationship between characteristics of their upbringing in direct comparison to adult activity levels (e.g., whether individuals were early or late laid or hatched). Nevertheless, the determinants of the experiment treatments and brood size on the level of activity during begging in the larger sample of nestlings that were investigated are detailed in the earlier study (Gilby, Sorato & Griffith, 2012). Here, we focused on the previously measured variables that were likely to be most reflective of mechanical speed and activity levels within a begging bout, namely Head movements and Tongue movements. ‘Head movements’ was defined as the average number of half turns of the nestling’s head made per second while the nestling was begging. ‘Tongue movements’ was defined as the average number of times the tongue moved from one side of the mouth to the other per second while the nestling was begging.

### Adult activity levels

Adult data was collected from the surviving adults in 2012 when the remaining 19 males and 16 females from the cross-fostering experiment were between 36 and 42 months old. The 35 surviving adults had been produced by 22 pairs and were reared in 20 foster nests (none were raised by their genetic parents). From August to December 2011, all of these individuals (that were now adults) were placed together in a single aviary (along with a number of other birds of the same age that had not been assayed as nestlings). They were provided with nest boxes and many of the adults paired up and bred. Individuals that bred together were considered to be a pair. Most of the individuals (15 of 19 males and 12 of 16 females) from the cross-fostering experiment paired up with a partner during this test. In April to May 2012, all birds were caught up from the aviary and the adults were measured (tarsus length and body mass), and placed in home cages (77 × 48 × 40 cm) inside. Each cage held a single pair, with seventeen pairs having formed naturally in the aviary and four of which were force-paired (by placing a random male and female together in a cage). All of these pairs were allowed to acclimate for a month in this home cage before the assay was conducted. Of the 21 pairs, there were seven where the data from only one of the individuals was included in this study, as that individual’s chosen or allocated partner had not been assayed as a nestling. The home cage activity test was conducted in June 2012. The home cage was provided with four perches (which were aligned in the same configuration for each cage at different angles and heights from 5 to 30 cm above the floor of the cage), a feeder, a grit container, a water container and a cuttlefish bone. At 12 pm on the day before the home cage test commenced, a sheet was placed over the home cage and it was transported to an isolated room (acoustically and visually from all other birds and stimuli) where the data would be collected. The pair were given the afternoon to adjust to their new surroundings (they remained in the same cage in which they had been held for the previous month). Just after dark, the room was discretely entered and filming was commenced with a video camera positioned two metres from the home cage (HDD/SD Card Hybrid Camcorder, SDR-H101; Panasonic, www.panasonic.com.au). The camera recorded video continuously for the entire next day, and was not collected again till after dark. The lights came on at 6:59 am and gradually increased to full brightness over 20 min, and began to dim again at 5:37 pm and went off at 5:57 pm. Total movements were recorded from the video data during the first 30 s of every 10 min period commencing when the lights came on at 6:59 am. A movement was defined as any movement greater than two body lengths, and consisted of either flights, or hops across the floor of the cage. This resulted in a total of 2310 observations recorded for the 35 individuals that completed the test.

### Ethical note

Animal welfare and methodological design were approved by the Animal Ethics Committee at Macquarie University (AEC reference number 2010/059). The health and condition of all birds were monitored on a daily basis and no individuals were noted to be unduly distressed by the experimental procedures or housing conditions.

### Statistical analyses

Due to sex differences in the consistencies in behaviours noted from previous studies (Mainwaring, Beal & Hartley, 2011; Schuett & Dall, 2009), the repeatability scores for males and females were analysed separately. Repeatability (*R*) values were calculated in R Development Core Team (2013) using the add-on package rptR, following the methods described by Nakagawa & Schielzeth (2010). Repeatability was calculated for the Head movements and Tongue movements begging variables over all six trials per individual. The repeatability of these variables was calculated for all of the nestlings that were raised in the original begging experiment (47 males and 51 females) and separately for just the 19 males and 16 females that survived to be later assayed in the home cage activity test. The repeatability of the Activity scores from the home cage activity test was calculated from all sixty 30-second intervals across the entire day excluding the first three and last three measurements taken, during which time individuals were in low level lighting and either waking up or preparing for sleep, and so generally more or less active than usual. This method of measuring repeatability differs from some other studies which measured behaviour only two or three times with days or weeks between measures and hence over a longer overall timeframe. However, we feel that the very high number of repeat measures (60 within the day) make up for our shorter overall timeframe. The data recorded for the Tongue movements, Head movements and Activity score variables were typical of count data following a Poisson distribution and so repeatability was calculated using the methods described for count data for these variables. These methods utilised multiplicative dispersion GLMMs (in which the overdispersion is modelled as a further parameter to the distribution from which the original responses are assumed to be derived) with a log link for estimating repeatability on the original scale (Nakagawa & Schielzeth, 2010). However, for the Tongue movements and Head movements variables these models were overdispersed indicating they performed poorly so we calculated these repeatabilities using a more flexible Gaussian REML method instead (this method involves fitting LMMs and using restricted maximum likelihood to estimate unbiased variance components) (Nakagawa & Schielzeth, 2010). Due to discrepancies between LRT *P* values and 95% confidence intervals (as is often the case when following these methods, the LRT *P* values were too small, (S Nakagawa, pers. comm., 2013)), *P* values were not reported. Instead, only the 95% CIs were reported and the interpretation focused on effect sizes of repeatability rather than their statistical significance (Nakagawa & Cuthill, 2007). Further analyses were conducted with the statistical software programme IBM SPSS v21.0 (Version 21.0; Armonk, NY). The mean scores from the begging trials conducted on day five and day seven were averaged to produce a single Head movements and Tongue movements score for each individual. An Activity score was calculated for each individual that completed the home cage activity test by dividing the total movements recorded overall by the number of 30 s periods analysed (*N* = 66). A generalised linear model utilising a binomial distribution with logit link was conducted to analyse the variation in survival of individuals that completed the begging assays to completion of the adult activity test. Head movements and Tongue movements were included as fixed covariates, with Sex as a fixed factorial term. The maximal model had all of the fixed effects and their two-way interaction terms and the model was rotated in a stepwise manner so each term was tested for significance when they were the last term in the model. Non-significant effects were dropped if their inclusion did not increase the explanatory power of the model until the minimal model was produced. A GLMM utilising a normal distribution with an identity link was performed to analyse the variation in adult activity levels, with covariates of Tongue movements, Head movements, Sex and the interactions of the former two variables with Sex. Foster nest identity, Genetic nest identity and Pair identity (in the adult activity test) were included as random effects. However, as this model did not converge properly, we conducted two separate GLMMs (using normal distribution with identity link) for Tongue movements and Head movements with the above-mentioned random effects included and Sex and the interaction with Sex included as fixed effects. Finally, we ran three separate multiple regressions to predict body mass from tarsus length and either adult activity level, nestling head movements, or nestling tongue movements.

## Results

**Figure 1:**
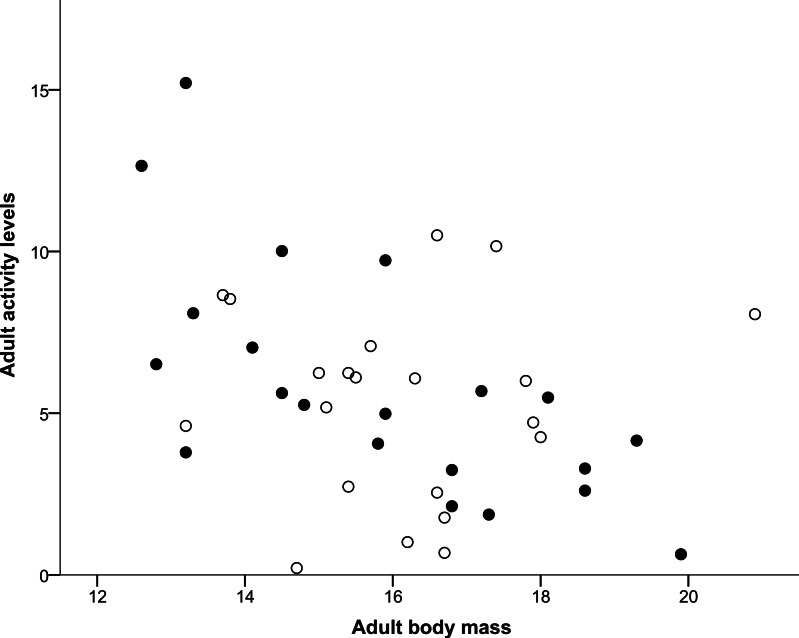
Correlation between adult body mass and adult activity levels. Males are represented with filled circles and females with open circles. ‘Adult body mass’ was measured in grams and ‘Adult activity levels’ was defined as the average number of movements made per 30-s period.

**Figure 2:**
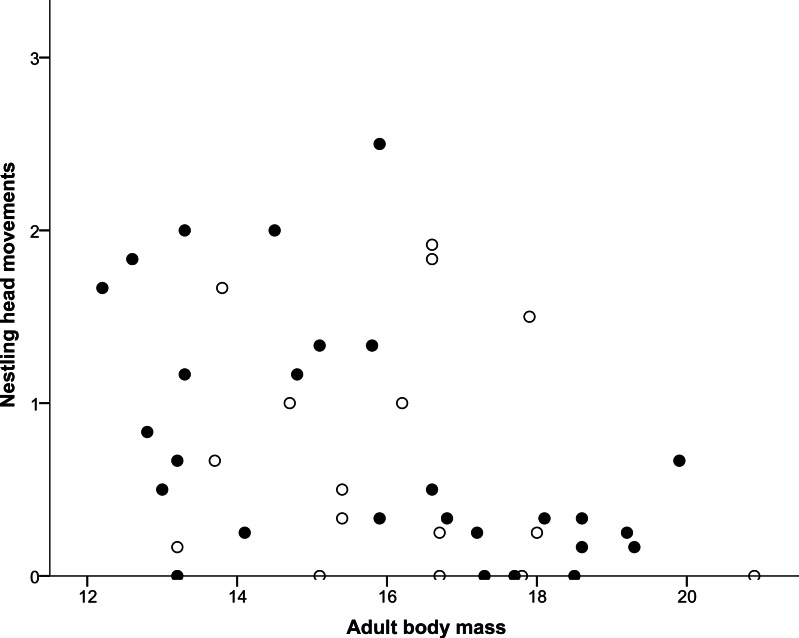
Correlation between adult body mass and nestling head movements. Males are represented with filled circles and females with open circles. ‘Adult body mass’ was measured in grams and ‘Nestling head movements’ was defined as the average number of half turns of the nestling’s head made per second.

Both nestling begging activity behaviours measured (Head movements and Tongue movements) were repeatable in males and females, both in the larger sample (all nestlings in the original experiment) and for the smaller set of individuals that completed the home cage activity test, as shown in Table 1. Repeatability estimates for these variables ranged from 0.372 for male tongue movements for the smaller set of individuals that completed the home cage activity test to 0.569 for male head movements again for the smaller set, with all other nestling repeatability estimates lying between these values. Head movements and tongue movements were significantly positively correlated with each other at both the observation (*r* = 0.633, *N* = 568, *P* < 0.0001) and individual level (*r* = 0.750, *N* = 98, *P* < 0.0001). In the home cage activity test, activity levels of adults were repeatable in males and females for the measurements made over the course of the day, also shown in Table 1, and ranged from 0.319 for males to 0.439 for females. As shown in Table 2, Sex was determined to be the most likely factor predicting whether an individual died during the period between the day 7 begging assay and the adult activity test when individuals were 36–42 months old, with females more likely to die during this period than males. In contrast, neither Tongue movements nor Head movements were found to significantly influence survival (Table 2). The mean number of head movements during begging was found to be significantly positively correlated with adult activity levels measured 36–42 months later (Table 3). However, the mean number of tongue movements was not significantly correlated with adult activity levels (Table 3). Moreover, as shown above, neither the sex of the focal individual nor the interactions between sex and either head or tongue movements were significantly correlated with adult activity levels in either model (Table 3). From a multiple regression with adult activity levels (*F*_2,38_ = 4.717, *P* = 0.015, *R*^2^ = 0.199), we found that adult activity levels predicted body mass (*P* = 0.007; Fig. 1) but tarsus length did not (*P* = 0.134). Similarly, from a multiple regression (*F*_2,39_ = 4.405, *P* = 0.019, *R*^2^ = 0.184), we found that head movements predicted body mass (*P* = 0.006; Fig. 2), but tarsus length did not (*P* = 0.833). However, from a further multiple regression (*F*_2,39_ = 1.731, *P* = 0.190, *R*^2^ = 0.082), we found that neither tongue movements (*P* = 0.077) nor tarsus length (*P* = 0.876) predicted body mass.

**Table 1:** Summary of repeatabilities. The repeatability (*R*) of individuals’ behaviour in the (a) nestling begging experiment and (b) home cage activity test. Repeatability in (a) was calculated from all of the six individual begging trials available for each individual. Repeatability of Activity score was calculated by comparing the middle 60 30-second movement scores over the course of the day.

Component	*R* ± SE	*N*	Lower CI	Upper CI
**(a) Nestling begging activity**				
**All nestlings**				
**Head movements**				
Male	0.466 ± 0.067	47	0.321	0.583
Female	0.492 ± 0.066	51	0.349	0.608
**Tongue movements**				
Male	0.514 ± 0.066	47	0.371	0.623
Female	0.47 ± 0.065	51	0.33	0.582
**Nestlings that matured only**				
**Head movements**				
Male	0.569 ± 0.102	19	0.329	0.729
Female	0.536 ± 0.114	16	0.292	0.715
**Tongue movements**				
Male	0.372 ± 0.109	19	0.136	0.555
Female	0.468 ± 0.119	16	0.196	0.665
**(b) Home cage activity test**				
**Activity score**				
Male	0.319 ± 0.083	19	0.149	0.469
Female	0.439 ± 0.104	16	0.21	0.615

**Table 2:** Summary of a Generalised Linear Model (GLM). The GLM examined how the sex of the focal bird and the mean number of head movements and tongue movements produced by nestlings during begging influenced survival to the home cage activity test.

Term	B	SE	DF	Wald Chi-Square	*P* value	Upper CI	Lower CI
Sex of focal bird	1.064 (female)	0.422	1	6.371	0.012	0.238	1.890
Tongue movements	0.101	0.263	1	0.147	0.702	−0.415	0.617
Head movements	0.027	0.456	1	0.004	0.953	−0.866	0.921
Tongue movements × sex of focal bird	−0.304 (female)	0.536	1	0.322	0.570	−1.355	0.746
Head movements × sex of focal bird	0.367 (female)	0.928	1	0.156	0.693	−1.451	2.184

**Table 3:** Summary of Generalised Linear Mixed Models (GLMMs). The GLMMs examined: (a) how the mean number of head movements and the sex of the focal bird influence adult activity levels during the home cage activity test and (b) how the mean number of tongue movements and the sex of the focal bird influence adult activity levels during the home cage activity test.

Term	Coefficient	SE (fixed) /SD (random)	DF	*F*-Value	*P* value	Upper CI	Lower CI
**(a) Head movements GLMM**							
Head movements	2.114	0.633	33	11.144	0.002	0.826	3.402
Sex of focal bird	1.025 (male)	0.829	32	1.528	0.225	−0.664	2.714
Head movements × sex of focal bird	1.157 (male)	1.418	31	0.666	0.421	1.735	4.049
Foster nest identity	6.238	2.498					
Genetic nest identity	1.756	1.325					
Pair identity	2.083	1.443					
**(b) Tongue movements GLMM**							
Tongue movements	1.335	0.810	33	2.717	0.109	−0.313	2.983
Sex of focal bird	1.140 (male)	0.992	32	2.020	0.165	−0.611	3.430
Tongue movements × sex of focal bird	1.905 (male)	1.390	31	1.878	0.180	−0.930	4.739
Foster nest identity	1.990	1.411					
Genetic nest identity	3.692	1.921					
Pair identity	1.722	1.312					

## Discussion

Our study confirms that the number of head and tongue movements made during nestling begging bouts were repeatable over time (in this study, roughly a 48-hour period) in zebra finch nestlings, providing evidence that ‘begging activity’ may be reflective of, or itself constitute, a personality variable that is measurable at a very young age. Moreover, the mean number of head movements, but not the mean number of tongue movements made during the nestling begging assays, was significantly positively correlated with adult activity levels measured much later in their life, several years later. This suggests that the tendency for individuals to be more active in different contexts persists over the majority of their lifespan, and is established very early in life. This is the first study in a bird to uncover evidence that nestling behaviour is predictive of long-term adult phenotype (but see Fucikova et al., 2009), and the first to compare a repeatable behavioural trait measured in very young birds with a similar trait much later in life. Previous work on the same species has demonstrated links between activity and traits such as boldness, aggression, neophobia, dominance, leadership and the use of producer/scrounger foraging tactics (Beauchamp, 2000; David, Auclair & Cézilly, 2011; David et al., 2012; David, Cézilly & Giraldeau, 2011; Martins et al., 2007; Schuett & Dall, 2009; Schuett, Dall & Royle, 2011). These correlations between personality traits imply the existence of what is often referred to as a behavioural syndrome in this species, whereby individuals tend to occupy one of two behavioural types: ‘fast/proactive’ or ‘slow/reactive’ (David, Auclair & Cézilly, 2011). This raises the possibility that nestling begging activity may potentially be used to predict not only adult phenotypes but also an individual’s behavioural type (‘fast/proactive’ versus ‘slow/reactive’ at a very young age), which if verified would open up further research possibilities for investigations of quantitative genetics and studies of selection on these traits.

It is unclear why we found a significant positive relationship between the mean number of nestling head movements during begging movements and adult activity levels, but failed to find a significant relationship between adult activity levels and the mean number of tongue movements. While both behaviours are thought to be produced to attract the attention of parents and provoke feeding (Kilner & Johnstone, 1997), it may be that the speed of movement of a large part of the body (the entire head and neck region) is more reflective of the general activity levels of an individual than the comparatively smaller tongue region. Sex was found to be a significant factor explaining whether or not an individual survived the 36–42 month period until the home cage activity test was conducted, with males more likely to survive than females, a result that is consistent with previous captive studies (Burley, 1985). However, sex did not significantly explain the significant relationship between nestling head movements and adult activity levels, which suggests that the positive relationship between the two variables is not sex-specific. In contrast, one factor that was significantly correlated with nestling and adult behaviour was adult body mass. Individuals with a comparatively higher body mass tended to make less head movements during begging as nestlings and tended to have lower activity levels as adults in the home cage activity test. Unfortunately, it is difficult to disentangle the cause and effect of this relationship, as individuals may differ in nestling head movements either due to intrinsic differences in activity or because they are trying to acquire more food via begging. A previous study found that food-rationed great tit nestlings tended to display higher exploratory behaviour as adults than control nestlings (Carere et al., 2005a), which suggests that receiving less food during ontogeny might produce differences in adult personality. We also found that adults with a lower body mass were more active. There are a number of reasons why individuals who are carrying excess weight would exhibit lower activity levels. Individuals might be following different energy storage tactics, with some individuals storing large amounts of energy as fat to preserve against starvation, and others maintaining little fat to remain light and agile (thereby making it easier to escape from predators). In this manner, general activity levels may be linked to the relationship between energetic gains and mortality, with different individuals employing different life-history tactics with regards to factors such as growth and fecundity (Stamps, 2007; Biro & Stamps, 2008). Moreover, the long-lasting link we found between general activity levels and body mass suggests that activity might be tied to individual differences in metabolic rate, especially as high levels of movements are energetically costly (Careau et al., 2008). Hence, we suggest that this inference should be addressed by future work that directly measures metabolic rate.

Due to the relatively low sample size of individuals that survived to the onset of our adult activity test, this study did not attempt to directly address whether ontogenetic factors, such as hatch order, yolk hormonal distribution, nutrition and the specifics of the social environment influenced adult personality and behaviour. Moreover, we did not include a non-fostered control group which limits our ability to measure the effects of environmental post-hatching parental influences. However, our results suggest that adaptations to environmental variation in early life (greater begging intensity and activity to compete with either older, or more well-fed nest mates) may persist into adulthood (Roulin, Dreiss & Kölliker, 2010). These adaptations may affect fitness, as activity has been shown to influence fitness attributes such as predator–prey interactions, resource acquisition and the risk of mortality (Sweeney et al., 2013; Werner & Anholt, 1993). Our findings suggest that the zebra finch may make a good candidate system for the study of the influences of ontogeny on adult personality. Being able to predict adult personality in very young offspring (in our study just 18 days after the formation of the zygote) will enable the examination of selection on these aspects of personality (as many individuals die in the first months of life), opening up new research opportunities in this field. Moreover, as it can be measured prior to significant episodes of selection, it will facilitate the study of the quantitative genetics of personality as well as its relationship to fitness.

## Supplemental Information

10.7717/peerj.566/supp-1 Supplemental Information 1 Supplemental raw data for both nestlings and adults

## References

[ref-1] Beauchamp G (2000). Individual differences in activity and exploration influence leadership in pairs of foraging zebra finches. Behaviour.

[ref-2] Biro PA, Stamps JA (2008). Are animal personality traits linked to life-history productivity?. Trends in Ecology & Evolution.

[ref-3] Brommer JE, Kluen E (2012). Exploring the genetics of nestling personality traits in a wild passerine bird: testing the phenotypic gambit. Ecology and Evolution.

[ref-4] Burley N (1985). Leg-band color and mortality patterns in captive breeding populations of zebra finches. The Auk.

[ref-5] Careau V, Thomas D, Humphries MM, Réale D (2008). Energy metabolism and animal personality. Oikos.

[ref-6] Carere C (2003). Personalities as epigenetic suites of traits: a study on a passerine bird. D. Phil. Thesis.

[ref-7] Carere C, Drent PJ, Koolhaas JM, Groothuis TGG (2005b). Epigenetic effects on personality traits: early food provisioning and sibling competition. Behaviour.

[ref-8] Carere C, Drent PJ, Privitera L, Koolhaas JM, Groothuis TGG (2005a). Personalities in great tits, *Parus major*: consistency and stability. Animal Behaviour.

[ref-9] Carere C, Maestripieri D (2013). Animal personalities: behavior, physiology, and evolution.

[ref-10] Cavigelli SA, McClintock MK (2003). Fear of novelty in infant rats predicts adult corticosterone dynamics and an early death. Proceedings of the National Academy of Sciences of the United States of America.

[ref-11] David M, Auclair Y, Cézilly F (2011). Personality predicts social dominance in female zebra finches, *Taeniopygia guttata*, in a feeding context. Animal Behaviour.

[ref-12] David M, Auclair Y, Cézilly F (2012). Assessing short- and long-term repeatability and stability of personality in captive zebra finches using longitudinal data. Ethology.

[ref-13] David M, Auclair Y, Giraldeau L-A, Cézilly F (2012). Personality and body condition have additive effects on motivation to feed in Zebra Finches *Taeniopygia guttata*. Ibis.

[ref-14] David M, Cézilly F, Giraldeau L-A (2011). Personality affects zebra finch feeding success in a producer–scrounger game. Animal Behaviour.

[ref-15] Eising C, Groothuis T (2003). Yolk androgens and begging behaviour in black-headed gull chicks: an experimental field study. Animal Behaviour.

[ref-16] Fucikova E, Drent PJ, Smits N, Van Oers K (2009). Handling stress as a measurement of personality in great tit nestlings (*Parus major*). Ethology.

[ref-17] Gilby AJ, Mainwaring MC, Griffith SC (2011). The adaptive benefit of hatching asynchrony in wild zebra finches. Animal Behaviour.

[ref-18] Gilby AJ, Sorato E, Griffith SC (2012). Maternal effects on begging behaviour: an experimental demonstration of the effects of laying sequence, hatch order, nestling sex and brood size. Behavioral Ecology and Sociobiology.

[ref-19] Heidinger BJ, Blount JD, Boner W, Griffiths K, Metcalfe NB, Monaghan P (2012). Telomere length in early life predicts lifespan. Proceedings of the National Academy of Sciences of the United States of America.

[ref-20] Herde A, Eccard J (2013). Consistency in boldness, activity and exploration at different stages of life. BMC Ecology.

[ref-21] Huff G, Huff W, Rath N, Donoghue A, Anthony N, Nestor K (2007). Differential effects of sex and genetics on behavior and stress response of turkeys. Poultry Science.

[ref-22] Kacelnik A, Cotton PA, Stirling L, Wright J (1995). Food allocation among nestling starlings: sibling competition and the scope of parental choice. Proceedings of the Royal Society of London Series B: Biological Sciences.

[ref-23] Kanda LL, Louon L, Straley K (2012). Stability in activity and boldness across time and context in captive Siberian dwarf hamsters. Ethology.

[ref-24] Kilner R, Johnstone RA (1997). Begging the question: are offspring solicitation behaviours signals of need?. Trends in Ecology & Evolution.

[ref-25] Mainwaring MC, Beal JL, Hartley IR (2011). Zebra finches are bolder in an asocial, rather than social, context. Behavioural Processes.

[ref-26] Martin JGA, Réale D (2008). Temperament, risk assessment and habituation to novelty in eastern chipmunks, *Tamias striatus*. Animal Behaviour.

[ref-27] Martins TLF, Roberts ML, Giblin I, Huxham R, Evans MR (2007). Speed of exploration and risk-taking behavior are linked to corticosterone titres in zebra finches. Hormones and Behavior.

[ref-28] Muller RE, Smith DG (1978). Parent-offspring interactions in zebra finches. The Auk.

[ref-29] Nakagawa S, Cuthill IC (2007). Effect size, confidence interval and statistical significance: a practical guide for biologists. Biological Reviews.

[ref-30] Nakagawa S, Schielzeth H (2010). Repeatability for Gaussian and non-Gaussian data: a practical guide for biologists. Biological Reviews.

[ref-31] Petelle MB, McCoy DE, Alejandro V, Martin JG, Blumstein DT (2013). Development of boldness and docility in yellow-bellied marmots. Animal Behaviour.

[ref-32] R Development Core Team (2013). R: a language and environment for statistical computing.

[ref-33] Réale D, Reader SM, Sol D, McDougall PT, Dingemanse NJ (2007). Integrating animal temperament within ecology and evolution. Biological Reviews.

[ref-34] Roulin A (2001). On the cost of begging vocalization: implications of vigilance. Behavioral Ecology.

[ref-35] Roulin A, Dreiss AN, Kölliker M (2010). Evolutionary perspective on the interplay between family life, and parent and offspring personality. Ethology.

[ref-36] Ruuskanen S, Laaksonen T (2010). Yolk hormones have sex-specific long-term effects on behavior in the pied flycatcher (*Ficedula hypoleuca*). Hormones and Behavior.

[ref-37] Ruuskanen S, Laaksonen T (2013). Sex-specific effects of yolk androgens on begging behavior and digestion in pied flycatchers. Journal of Avian Biology.

[ref-38] Schuett W, Dall SRX (2009). Sex differences, social context and personality in zebra finches, *Taeniopygia guttata*. Animal Behaviour.

[ref-39] Schuett W, Dall SRX, Royle NJ (2011). Pairs of zebra finches with similar ‘personalities’ make better parents. Animal Behaviour.

[ref-40] Seyfarth RM, Silk JB, Cheney DL (2012). Variation in personality and fitness in wild female baboons. Proceedings of the National Academy of Sciences of the United States of America.

[ref-41] Stamps JA (2007). Growth-mortality tradeoffs and ‘personality traits’ in animals. Ecology Letters.

[ref-42] Sweeney K, Cusack B, Armagost F, O’Brien T, Keiser CN, Pruitt JN (2013). Predator and prey activity levels jointly influence the outcome of long-term foraging bouts. Behavioral Ecology.

[ref-43] Tschirren B, Rutstein AN, Postma E, Mariette M, Griffith SC (2009). Short-and long-term consequences of early developmental conditions: a case study on wild and domesticated zebra finches. Journal of Evolutionary Biology.

[ref-44] Vennerholm L (2012). Ontogeny of personality in red junglefowl chicks, *Gallus gallus*. Student Thesis.

[ref-45] Von Engelhardt N, Carere C, Dijkstra C, Groothuis TGG (2006). Sex specific effects of yolk testosterone on offspring survival, begging and growth in the zebra finch. Proceedings of the Royal Society B: Biological Sciences.

[ref-46] Werner EE, Anholt BR (1993). Ecological consequences of the trade-off between growth and mortality rates mediated by foraging activity. American Naturalist.

[ref-47] Wilson ADM, Krause J (2012). Personality and metamorphosis: is behavioral variation consistent across ontogenetic niche shifts?. Behavioral Ecology.

[ref-48] Zann RA (1996). The zebra finch: a synthesis of field and laboratory studies.

